# Supplementary material for: Resolving the full spectrum of human genome variation using Linked-Reads
Source: Genome Res. 2019 Apr;29(4):635–45. doi: 10.1101/gr.234443.118 (PMC6442396; doi:10.1101/gr.234443.118)
Supplement: Supplemental Material [file supp_gr.234443.118_Supplemental_longranger-2.2.zip › longranger-2.2.tar.bz2!/lr2.2-public/lib/stan/cmdstan/stan/doc/api/html/functions_func_g.html]

Stan: Class Members - Functions


|  |  |
| --- | --- |
|  | Stan  2.5.0  probability, sampling & optimization |


- Main Page
- Related Pages
- Namespaces
- Classes
- Files

- Class List
- Class Index
- Class Hierarchy
- Class Members

- All
- Functions
- Variables
- Typedefs
- Enumerator
- Related Functions

- a
- b
- c
- d
- e
- f
- g
- h
- i
- j
- k
- l
- m
- n
- o
- p
- q
- r
- s
- t
- u
- v
- w
- x
- z
- ~


All Classes Namespaces Files Functions Variables Typedefs Enumerations Enumerator Friends Macros Pages

### - g -

- generate\_begin\_for\_dims()
  : stan::gm::validate\_var\_decl\_visgen
- generate\_buffer\_loop()
  : stan::gm::generate\_init\_visgen
- generate\_check\_double()
  : stan::gm::generate\_init\_visgen
- generate\_check\_int()
  : stan::gm::generate\_init\_visgen
- generate\_csv\_header\_array()
  : stan::gm::write\_csv\_header\_visgen
- generate\_declaration()
  : stan::gm::generate\_init\_visgen
- generate\_dims\_array()
  : stan::gm::write\_dims\_visgen
- generate\_dims\_loop\_fwd()
  : stan::gm::generate\_init\_visgen
- generate\_end\_for\_dims()
  : stan::gm::validate\_var\_decl\_visgen
- generate\_increment()
  : stan::gm::set\_param\_ranges\_visgen
- generate\_increment\_i()
  : stan::gm::set\_param\_ranges\_visgen
- generate\_indent\_num\_dims()
  : stan::gm::generate\_init\_visgen
- generate\_init()
  : stan::gm::generate\_local\_var\_init\_nan\_visgen
- generate\_init\_args()
  : stan::gm::local\_var\_decl\_visgen
- generate\_init\_vars\_visgen()
  : stan::gm::generate\_init\_vars\_visgen
- generate\_init\_visgen()
  : stan::gm::generate\_init\_visgen
- generate\_initialize\_array()
  : stan::gm::init\_local\_var\_visgen
  , stan::gm::write\_array\_visgen
  , stan::gm::write\_csv\_visgen
- generate\_initialize\_array\_bounded()
  : stan::gm::init\_local\_var\_visgen
  , stan::gm::write\_array\_visgen
  , stan::gm::write\_csv\_visgen
- generate\_local\_var\_init\_nan\_visgen()
  : stan::gm::generate\_local\_var\_init\_nan\_visgen
- generate\_loop\_var()
  : stan::gm::validate\_var\_decl\_visgen
- generate\_name\_dims()
  : stan::gm::generate\_init\_visgen
- generate\_param\_names()
  : stan::gm::write\_param\_names\_visgen
- generate\_param\_names\_array()
  : stan::gm::constrained\_param\_names\_visgen
  , stan::gm::unconstrained\_param\_names\_visgen
- generate\_type()
  : stan::gm::local\_var\_decl\_visgen
- generate\_void\_statement()
  : stan::gm::local\_var\_decl\_visgen
- generate\_write\_loop()
  : stan::gm::generate\_init\_visgen
- get()
  : stan::gm::variable\_map
- get\_base\_type()
  : stan::gm::variable\_map
- get\_code\_string()
  : stan::optimization::BFGSMinimizer< FunctorType, QNUpdateType, Scalar, DimAtCompile >
- get\_covar\_adaptation()
  : stan::mcmc::stepsize\_covar\_adapter
- get\_current\_stepsize()
  : stan::mcmc::base\_hmc< M, P, H, I, BaseRNG >
- get\_delta()
  : stan::mcmc::stepsize\_adaptation
- get\_gamma()
  : stan::mcmc::stepsize\_adaptation
- get\_kappa()
  : stan::mcmc::stepsize\_adaptation
- get\_L()
  : stan::mcmc::base\_static\_hmc< M, P, H, I, BaseRNG >
- get\_max\_delta()
  : stan::mcmc::base\_nuts< M, P, H, I, BaseRNG >
- get\_max\_depth()
  : stan::mcmc::base\_nuts< M, P, H, I, BaseRNG >
- get\_mu()
  : stan::mcmc::stepsize\_adaptation
- get\_nominal\_stepsize()
  : stan::mcmc::base\_hmc< M, P, H, I, BaseRNG >
- get\_num\_dims()
  : stan::gm::variable\_map
- get\_origin()
  : stan::gm::variable\_map
- get\_param\_names()
  : stan::mcmc::ps\_point
- get\_params()
  : stan::mcmc::ps\_point
- get\_qnupdate()
  : stan::optimization::BFGSMinimizer< FunctorType, QNUpdateType, Scalar, DimAtCompile >
- get\_result\_type()
  : stan::gm::function\_signatures
- get\_sample\_param\_names()
  : stan::mcmc::sample
- get\_sample\_params()
  : stan::mcmc::sample
- get\_sampler\_diagnostic\_names()
  : stan::mcmc::base\_hmc< M, P, H, I, BaseRNG >
  , stan::mcmc::base\_mcmc
- get\_sampler\_diagnostics()
  : stan::mcmc::base\_hmc< M, P, H, I, BaseRNG >
  , stan::mcmc::base\_mcmc
- get\_sampler\_param\_names()
  : stan::mcmc::base\_mcmc
  , stan::mcmc::base\_nuts< M, P, H, I, BaseRNG >
  , stan::mcmc::base\_static\_hmc< M, P, H, I, BaseRNG >
- get\_sampler\_params()
  : stan::mcmc::base\_mcmc
  , stan::mcmc::base\_nuts< M, P, H, I, BaseRNG >
  , stan::mcmc::base\_static\_hmc< M, P, H, I, BaseRNG >
- get\_signature\_matches()
  : stan::gm::function\_signatures
- get\_stepsize\_adaptation()
  : stan::mcmc::stepsize\_adapter
  , stan::mcmc::stepsize\_covar\_adapter
  , stan::mcmc::stepsize\_var\_adapter
- get\_stepsize\_jitter()
  : stan::mcmc::base\_hmc< M, P, H, I, BaseRNG >
- get\_T()
  : stan::mcmc::base\_static\_hmc< M, P, H, I, BaseRNG >
- get\_t0()
  : stan::mcmc::stepsize\_adaptation
- get\_var\_adaptation()
  : stan::mcmc::stepsize\_var\_adapter
- grad()
  : stan::agrad::var
  , stan::optimization::BFGSLineSearch< M, QNUpdateType, Scalar, DimAtCompile >
- grad\_evals()
  : stan::optimization::BFGSLineSearch< M, QNUpdateType, Scalar, DimAtCompile >
- grad\_norm()
  : stan::optimization::BFGSLineSearch< M, QNUpdateType, Scalar, DimAtCompile >


---

[ Stan Home Page ]

*© 2011–2014,
Stan Development Team.*
